# Neonicotinoid-containing insecticide disruption of growth, locomotion, and fertility in *Caenorhabditis elegans*

**DOI:** 10.1371/journal.pone.0238637

**Published:** 2020-09-09

**Authors:** Beatrix R. Bradford, Elizabeth Whidden, Esabelle D. Gervasio, Paula M. Checchi, Kathleen M. Raley-Susman

**Affiliations:** 1 Department of Biology, Marist College, Poughkeepsie, New York, United States of America; 2 Department of Biology, Vassar College, Poughkeepsie, New York, United States of America; University of Louisville School of Medicine, UNITED STATES

## Abstract

Neonicotinoids, a class of insecticides structurally similar to nicotine that target biting and sucking insects, are the most widely used insecticides today, in part due to their supposed low toxicity in other organisms. However, a growing body of research has found that even low doses of neonicotinoids can induce unexpected negative effects on the physiology and survival of a wide range of non-target organisms. Importantly, no work has been done on the commercial formulations of pesticides that include imidacloprid as the active ingredient, but that also contain many other components. The present study examines the sublethal effects of “Tree and Shrub”™ (“T+S”), a commercial insecticide containing the neonicotinoid imidacloprid as its active ingredient, on *Caenorhabditis elegans*. We discovered that “T+S” significantly stunted the overall growth in wildtype nematodes, an effect that was exacerbated by concurrent exposure to heat stress. “T+S” also negatively impacted fecundity as measured by increased germline apoptosis, a decrease in egg-laying, and fewer viable offspring. Lastly, exposure to “T+S” resulted in degenerative changes in nicotinic cholinergic neurons in wildtype nematodes. As a whole, these findings demonstrate widespread toxic effects of neonicotinoids to critical functions in nematodes.

## Introduction

Neonicotinoids, a relatively new class of insecticides, are the most widely used insecticides today [1]. They act systemically and become distributed through all plant tissues. Neonicotinoids are widely applied as seed dressings and also are sprayed and applied topically. In most arthropods, neonicotinoids function as neurotoxins, acting as agonists of the nicotinic acetylcholine receptors (nAChRs) [2]. Binding of neonicotinoids to nAChRs is irreversible and induces over-stimulation leading to paralysis and eventual death. Neonicotinoids differ from nicotine in that they contain an electronegative moiety consisting of a either a nitroimine, cyanoimine, or nitromethylene pharmacophore that is presumed to selectively bind to the specific amino acids present in the loop structures of the ligand binding domain of insect nAChRs [3]. Because of this, these compounds are marketed as having no effect in vertebrate organisms and supposedly provide effective and selective pest control with many uses in farming and horticulture.

However, even low doses of neonicotinoids have negative effects on the physiology and survival of a wide range of non-target organisms including bees and vertebrates in terrestrial habitats [4,5]. A recent report described songbird death after ingestion of imidacloprid-coated seeds from drench-application to the base of trees in a residential neighborhood in California [6]. In addition to non-target organism toxicity from unanticipated exposure and ingestion, neonicotinoids can leach from soil into waterways and accumulate in soils [7], and they are found in the nectar and pollen of even untreated plants. Levels of some neonicotinoids in soils, waterways, field margin plants, and floral resources have been found at concentrations exceeding the insecticide’s LC_50_ for non-target organisms [4].

Imidacloprid is a systemic neonicotinoid insecticide that irreversibly binds to the insect nicotinic acetylcholine (ACh) receptor, leading to ACh accumulation and paralysis [1]. Because of its supposed low toxicity in non-arthropod animals, imidacloprid is used in many commercially-available insecticides to target sucking insects and is now the most commonly used neonicotinoid worldwide [8,9]. The half-life of imidacloprid is 1,250 days and it has been shown to accumulate over time in soil [1]. “Tree and Shrub”™ (“T+S”) is a commercially available insecticide marketed by Bayer with imidacloprid as its active ingredient at a concentration of 1.47% (w/v) and is the focus of this study. We chose to test directly the effects of this commercial preparation because many other compounds, called “inert ingredients,” are present that could interact with imidacloprid biologically or that could have biological effects on their own.

*Caenorhabditis elegans* is a free-living, non-parasitic nematode found in soils worldwide that is also well-known for its extensive use as a model organism important in the fields of developmental biology, neurobiology and genetics [10]. This species plays important roles in soil ecology by contributing to soil fertility and pathogen control [11]. Importantly, *C*. *elegans* is an established model for predicting chemical potency and biological outcomes of toxin exposure in mammals [12].

One notable reaction to environmental pollutants is an increase in reactive oxygen species (ROS) [13]. A recent study on thiacloprid, a neonicotinoid closely related to imidacloprid, revealed that this insecticide increased the expression of genes involved in apoptosis and cell secretion in *C*. *elegans* [14]. This study also found that heat and oxidative stress acted synergistically with thiacloprid to reduce overall survival, and that simultaneous sub-lethal exposure to heat and oxidative stress reduced the expression of several oxidative-stress response genes [14]. Additionally, several studies in zebrafish and rats have found that chronic exposure to imidacloprid induces oxidative damage in vertebrates [15,16]. Thus, we also examined whether exposure to “T+S” during heat stress would exacerbate the growth defects during late larval development of nematodes.

Of the 302 neurons present in the nervous system of adult *C*. *elegans* hermaphrodites, approximately 120 are cholinergic [17]. The nicotinic acetylcholine receptors (nAChRs) are located along the body-wall muscle and are responsible for controlling muscle contraction and therefore movement [18,19]. Accordingly, nAChRs also regulate egg-laying, one of the simplest motor actions performed by *C*. *elegans*. This behavior is modulated by various environmental cues and is controlled by multiple cholinergic motor neurons [18]. Exposure to nicotine decreases the expression of the cholinergic receptors at the vulva, leading to a decrease in egg laying [19]. Exposure to imidacloprid significantly reduced egg-laying and sperm viability in honeybees, suggesting that this neonicotinoid has a similar effect on fecundity as nicotine [20,21]. Pesticide use has affected fecundity of nematodes, exerting germline apoptosis during gonad development [22] and induces meiotic dysfunction in the adult germ line [23,24]. Thus, we also examined germline apoptosis, as well as brood size, as additional measures for effects on fecundity.

To date, there are no comprehensive studies examining the effects of imidacloprid-containing pesticide formulations on non-target organisms in a preparation available for public use. This is significant, as like other commercially-available pesticides, safety assessments are limited to their active ingredient(s), thus ignoring adjuvants and/or the mixture as a whole. Because organisms are exposed to the active ingredient when mixed with many other substances in a commercial formulation, it is critical to evaluate the effects of exposure to the full mixture, rather than just the active agent. Here, we present the first analysis of an imidacloprid-containing mixture on a non-target organism used as a model for both biomedical and environmental toxicology.

## Results

### “T+S” exposure stunts growth of *C*. *elegans*, an effect exacerbated by simultaneous heat stress

**Fig 1:**
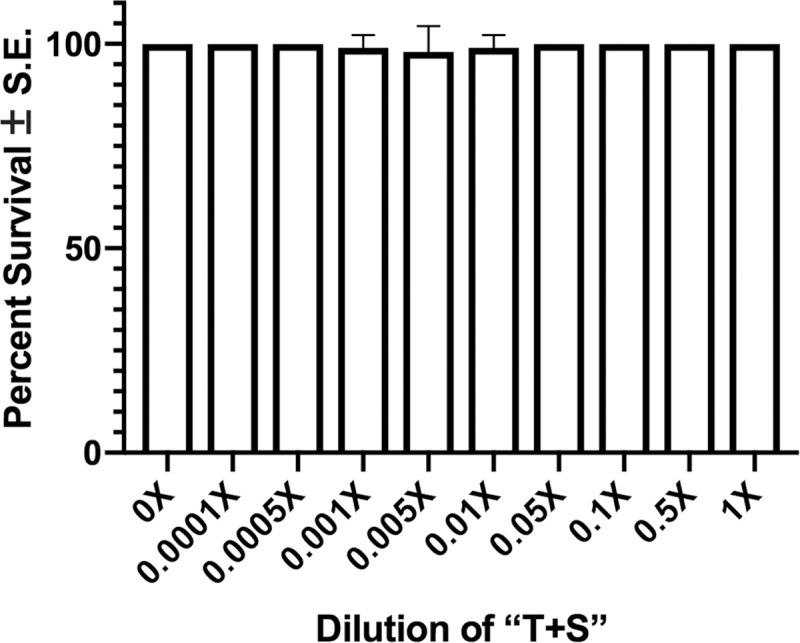
“T+S” is not lethal to *C*. *elegans*. Early L4 wildtype nematodes were exposed to different concentrations of “T+S” for 48 hours. Worms were then individually examined at 20X magnification and gently nudged with a sterilized platinum wire to induce motion. Immobile animals were presumed dead. Data from 10 biological replicates were analyzed using a 2-way ANOVA and pairwise *t*-tests with Bonferroni correction. (F_9, 890_ = 5.9 e^5^, *p =* 0.4798).

**Fig 2:**
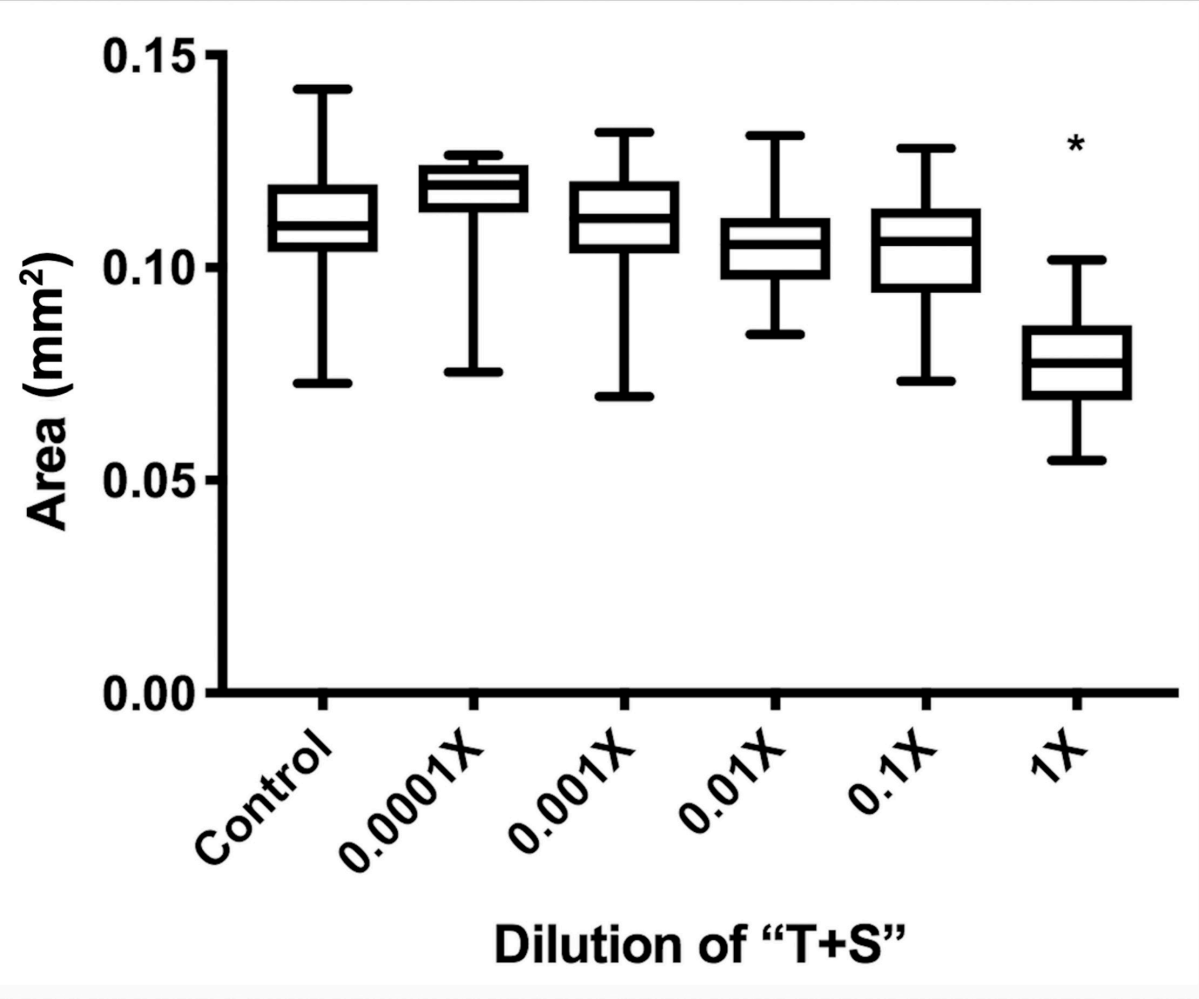
Growth of *C*. *elegans* is stunted by exposure to “T+S”. Area of *C*. *elegans* was measured in young adult, day 5 nematodes exposed to varying concentrations of “T+S” from laid egg to adulthood (day 5) to assess effects on growth and body size. *C*. *elegans* were imaged and then measured using Image J/FIJI software. n = 6 independent trials with 10 worms at each concentration (60 worms for each concentration total) were analyzed using a 2-way ANOVA. Area: F_5,136_ = 13.14, *p<*0.0001. t-test: control vs. 1X: *p<*0.0001.

**Fig 3:**
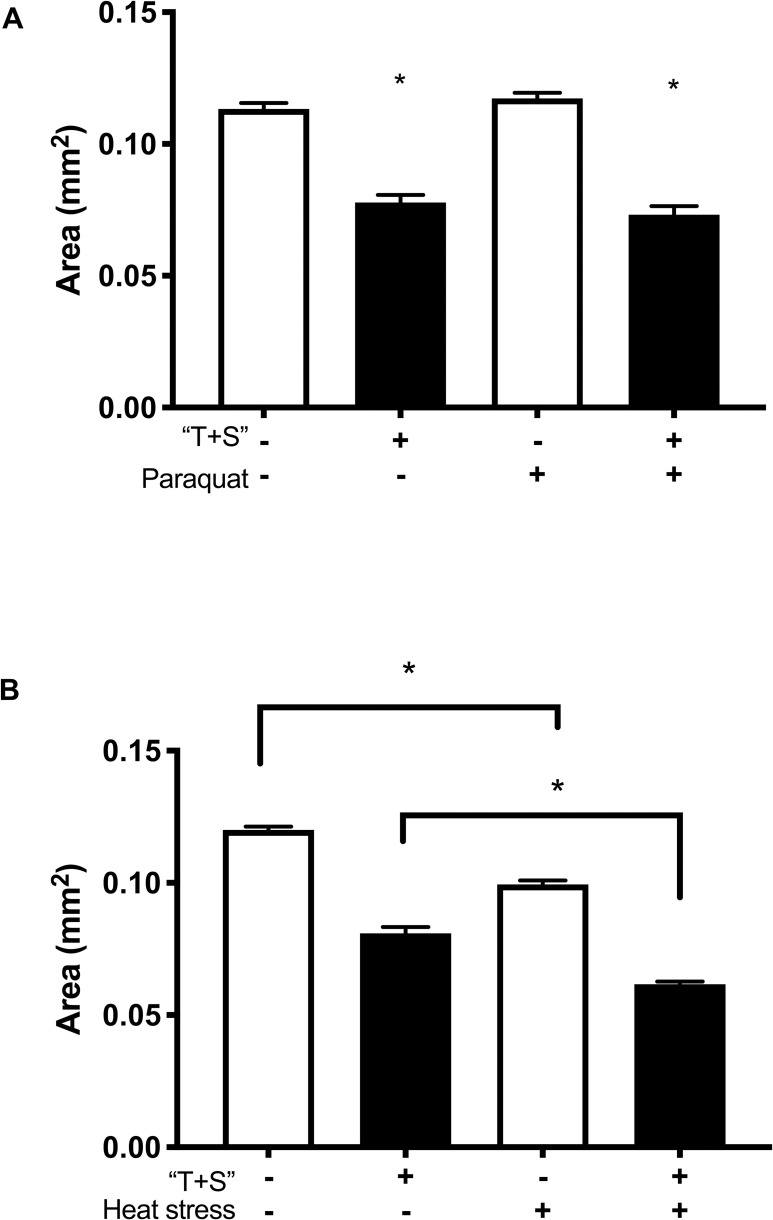
Growth is further stunted by combined exposure to heat stress and “T+S”. Cross-sectional area of day 5 *C*. *elegans* raised on “T+S” or control plates was measured 8 hours after exposure to either paraquat (a known oxidative stressor), 35˚C heat stress, or a water control. *C*. *elegans* were imaged and then measured using Image J/FIJI software. A total of 36–64 worms were analyzed for each treatment group or control. Data were analyzed using a 1-way ANOVA. “T+S” significantly reduced body size (*p<*0.0001), as did heat stress (*p<*0.0001). Heat stress significantly increased the effect of “T+S” (n = 190, *p<*0.001). In contrast, paraquat did not affect body size (n = 122, *p =* 0.93) and the combination of both “T+S” and paraquat had no additional effect (*p =* 0.1911).

A 48-hour exposure to “T+S” was not lethal to *C*. *elegans* at any tested concentration, including full strength (F_9, 890_ = 5.9 e^5^, *p =* 0.4798; Fig 1) when nematodes were exposed on petri plates. Nonetheless, there were numerous effects indicating toxicity of the pesticide. Full-strength “T+S” (the recommended concentration for application) was found to significantly reduce body size (F_5,136_ = 13.14, *p<*0.0001) of adult day-5 worms when analyzed using a one-way ANOVA (Fig 2). While paraquat, a source of oxidative stress, did not affect body size, heat stress significantly reduced body size (Fig 3). Intriguingly, heat stress significantly exacerbated the effects of “T+S” (n = 190, *p<*0.0001), while this did not occur with simultaneous exposure with paraquat, a known oxidative agent (n = 122, *p* = 0.93, Fig 3).

### Chronic, but not acute, “T+S” exposure decreases locomotion

**Fig 4:**
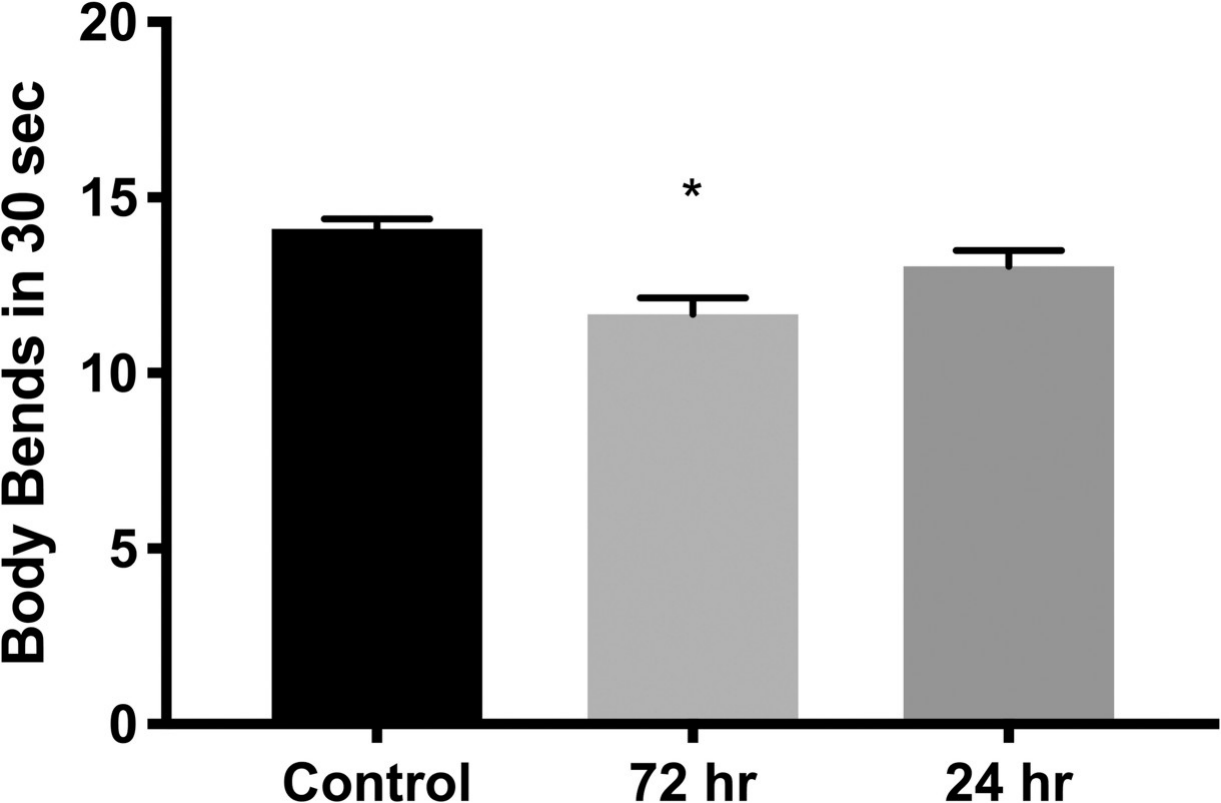
Locomotion is impaired by exposure to “T+S”. Total number of full sinusoidal body bends performed by individual worms in 30 seconds. Worms were raised on standard NGM plate plates for 72 hours to early L4 stage (“T+S from egg”) and then either transferred to new control plates (“Control”) or 1X “T+S” (“Treatment”) for 72 hours to early L4 stage. L4 worms raised on control plates for 72 hours were transferred to new control plates or ones pretreated with 1X “T+S” for 24 hours. L4 worms raised on plates pretreated with “T+S” for 72 hours were transferred to control plates for 24 hours. “T+S” was found to significantly decrease locomotion when worms were exposed to “T+S” from egg stage, but not from young L4 stage (“T+S from L4”) (*p* = 0.0003, 0.5793, respectively). Data shown are average ± SEM of 45–100 worms in each group; *p<0.001 when compared with control using one-way ANOVA.

L4 worms (96 hours old raised at 20°C with standard OP50 *E*. *coli* food source) raised on 1X “T+S” for 72 hours (from egg to L4) and then placed on control plates for 24 hours, showed decreased locomotion, as measured by the number of full sinusoidal body bends completed in 30 seconds (n = 135, *p =* 0.0003, Fig 4). However, this effect was not found in worms exposed for 24 hours beginning at the young L4 stage (n = 132, *p =* 0.5793).

### “T+S” impairs multiple aspects of reproduction

**Fig 5:**
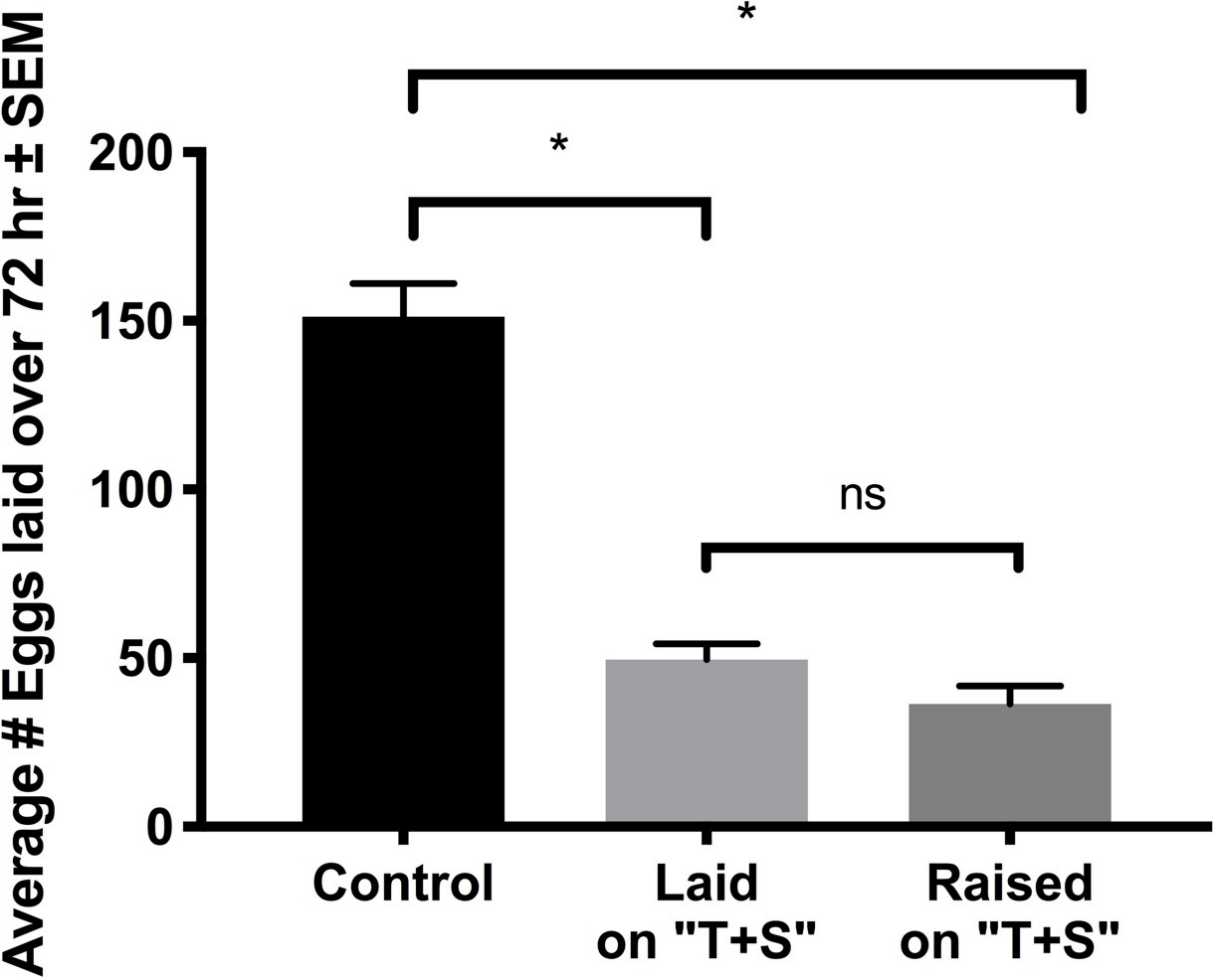
Egg-laying is reduced in “T+S” exposed nematodes. Total eggs laid over a three-day period by L4 worms. Worms were raised in either standard NGM plate (“Control”) or ones treated with 1X “T+S” to the L4 stage (“Raised”) and then transferred to “T+S” plates for the egg-laying period (“Laid” and “Raised”). “T+S” was found to significantly decrease the number of eggs laid, both when worms laid eggs on “T+S” and when they were raised on “T+S”. (*p<*0.0001 for control vs. both treatment groups, *p =* 0.3794 for treatment groups).

Exposure to “T+S” reduced the number of eggs laid by young adult worms over a three-day period (Fig 5). This was the case for worms raised to the L4 stage on control plates and moved to NGM plates treated with “T+S” to lay eggs (n = 37, *p<*0.0001), and with worms raised to the L4 stage on “T+S” plates and transferred to “T+S” plates to lay eggs (n = 35, *p<*0.0001).

**Fig 6:**
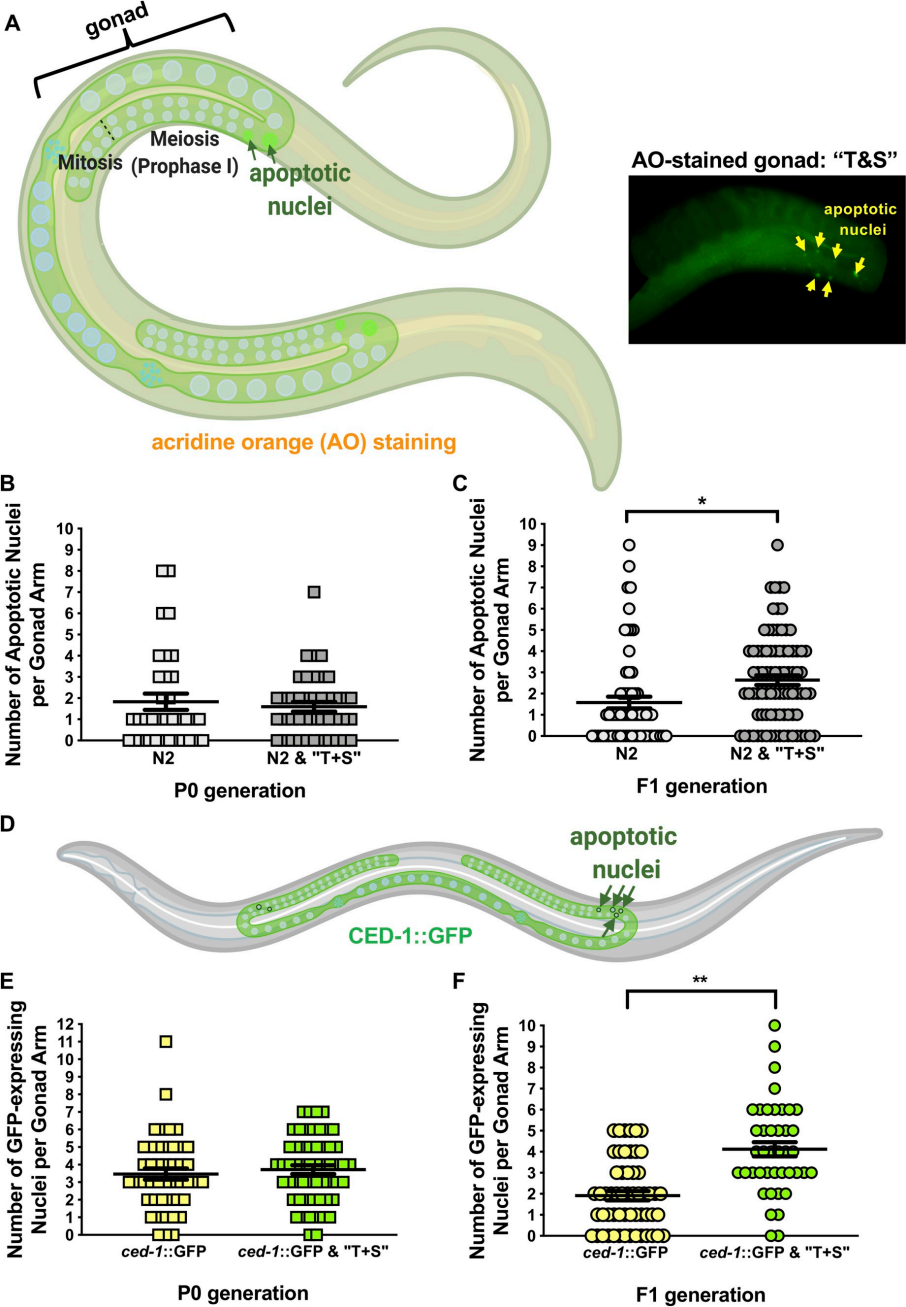
“T+S” exposure results in elevated germline apoptosis in the offspring of wild-type germ lines. Wild-type germ lines were assessed using acridine orange (AO) (A-C), an indicator of pH change in apoptotic nuclei, and CED-1::GFP (D-F), a tagged protein expressed in apoptotic cells. (A) Left: Graphic depicting *C*. *elegans* stained with AO. Arrows indicate region of meiosis where apoptotic bodies are typically detected. Right: Representative AO-stained gonad from offspring of a live, N2 hermaphrodite exposed to “T&S.” Image is a projection of merged Z-stacks and is cropped to highlight the germ line. Nuclei that accumulate AO (yellow arrowheads) are detected in late pachytene/diplotene of prophase I. (B-C) Apoptotic nuclei of wild-type N2 germ lines were quantified using AO in parents (P0, B) and their offspring (F1, C) in the presence and absence of pesticide treatment. (D) Graphic depicting wild-type *C*. *elegans* expressing *ced-1*::GFP. Green “circles” depict phagocytic nuclei, which are subsequently cleared by apoptosis. (D-E) Quantification of apoptosis using *ced-1*::GFP-expression in parents (P0, D) and their offspring (F1, E) in the presence and absence of pesticide treatment. Each data point (i.e. shape) corresponds to total number of apoptotic nuclei per gonad arm from a single animal. Number of gonads scored per P0 genotype are as follows: wild-type (N2) control, n = 35; wild-type (N2) “T+S”, n = 42; *ced-1*::GFP control, n = 45; *ced-1*::GFP “T+S”, n = 56. Number of gonads scored per F1 genotype are as follows: wild-type (N2) control, n = 64; wild-type (N2) “T+S”, n = 85; *ced-1*::GFP control, n = 56; *ced-1*::GFP “T+S”, n = 42. Each corresponding data set is representative of a minimum of 3 independent trials. Significance for all data sets was determined by a two-tailed Mann Whitney test. Horizontal lines correspond to the mean for each data set. Error bars = S.E.M. *denotes *p<*0.001 and **denotes *p≤*0.0001. Graphics were created with BioRender.

Defects in egg laying behavior are associated with both neural and reproductive dysfunction [25]. As egg-laying defects are coincident with increased germline apoptosis in other studies examining pesticide exposure in worms [22,26], apoptotic nuclei were quantified in the germ lines of live, wild-type adults exposed to “T+S” for 48 hours (Fig 6). Though P0 generation animals were unaffected, we found a significant increase in germline apoptosis in their offspring (Fig 6B and 6C, *p =* 0.0003). As a complementary assay, we measured germline apoptosis in live *ced-1*::GFP worms, a transgenic strain expressing a transmembrane receptor required for engulfment of germ cell corpses [26; Fig 6D]. Compared to other methods such as acridine orange, the CED-1::GFP fusion protein is reportedly a more sensitive means of assessing germline apoptosis [27]. Similar to wild-type (N2) animals, there was no change in the number of apoptotic nuclei in the P0 exposed *ced-1*::GFP worms while a significant increase in germ cell corpses in the F1 germline in response to “T+S” exposure (Fig 6E and 6F, *p<*0.0001).

**Fig 7:**
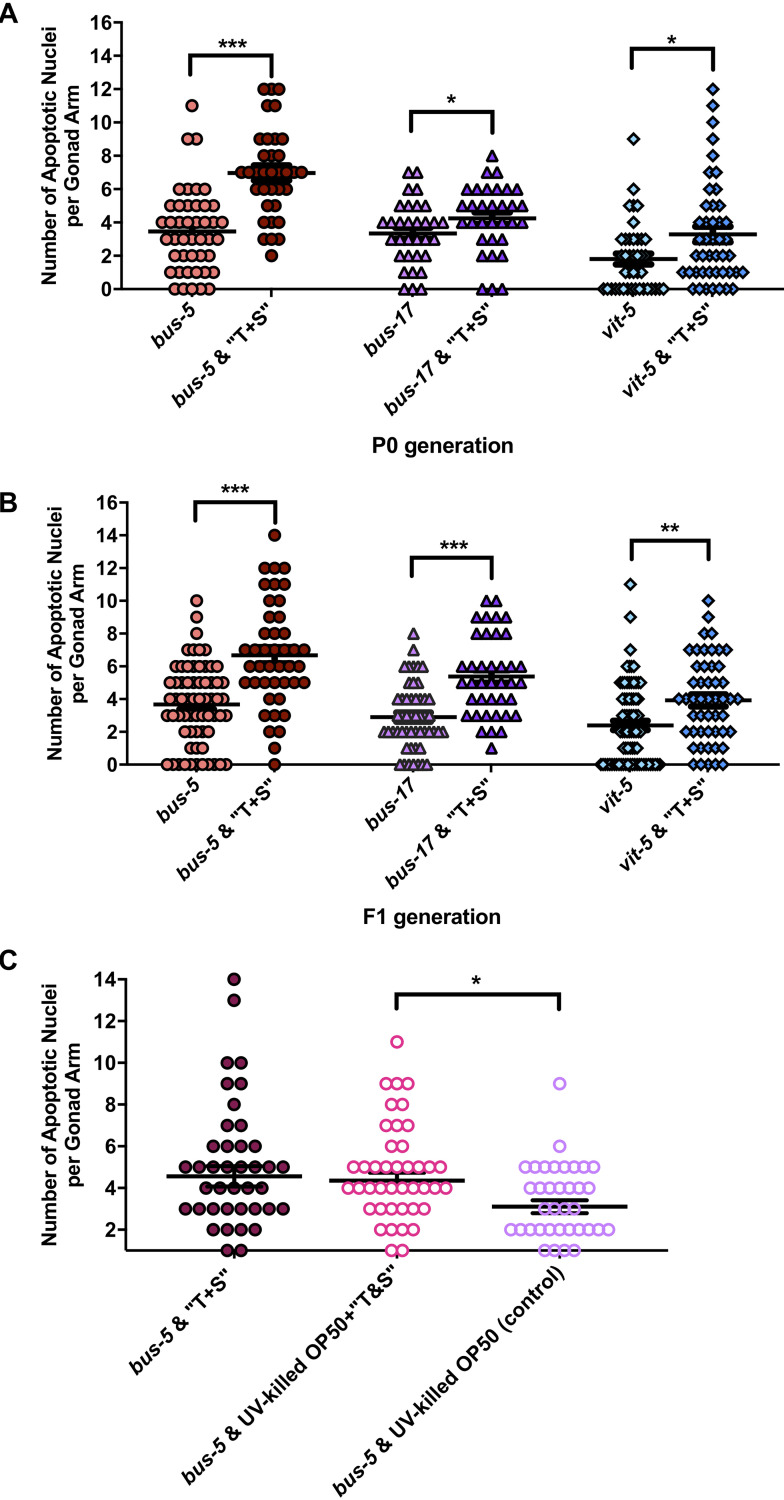
“T+S” increases germline apoptosis in P0 and F1 generations in sensitized strains of *C*. *elegans*. Germline apoptosis was assessed using acridine orange in (A) P0 and (B) F1 generation worms possessing the following mutations: *bus-*5, *bus-17* and *vit-*5. Each data point (i.e. shape) corresponds to total number of apoptotic nuclei per gonad arm from a single animal. Number of gonads scored per P0 genotype are as follows: *bus-5* control, n = 44; *bus-5* “T+S”, n = 35; *bus-17* control, n = 32; *bus-17* “T+S”, n = 32; *vit-5* control, n = 41; *vit-5* “T+S”, n = 45. Number of gonads scored per F1 genotype are as follows: *bus-5* control, n = 65; *bus-5* “T+S”, n = 43; *bus-17* control, n = 51; *bus-17* “T+S”, n = 39; *vit-5* control, n = 77; *vit-5* “T+S”, n = 50. Each data set is representative of a minimum of three biological replicates. (C) Quantification of germline apoptosis in the P0 generation of *bus-5* mutants exposed to “T+S” combined with living versus UV-killed *E*. *coli*. Data are representative of two independently-performed trials, and n values are as follows: *bus-5* “T+S”, n = 46; *bus-5* “T+S” + UV-killed OP50 *E*.*coli*, n-46; *bus-5* + UV-killed OP50 *E*.*coli* (control), n = 39. For all experiments, significance was determined by a two-tailed Mann Whitney test. Horizontal lines correspond to the mean for each data set. Error bars = S.E.M. *denotes *p<*0.05; **denotes *p<*0.001 and ***denotes *p≤0*.0001.

Next, we scored apoptotic nuclei in three sensitized strains: two cuticle-permeable strains, *bus-5* and *bus-17* [28], and *vit-5* mutants, which lack a vitellogenin protein known to mediate toxic stress response following exposure to organophosphates [29]. Whereas increased apoptosis was limited to the F1 progeny of wild-type animals exposed to “T+S” (Fig 6), the germ lines of both cuticle-defective strains and *vit-5* mutants contained significantly increased apoptotic bodies in both the P0 and F1 generation (Fig 7A and 7B). To test the specificity of pesticide exposure, we assessed germline apoptosis in *bus-5* mutants fed UV-killed *E*. *coli*. Apoptosis remained elevated in UV-killed *E*. *coli* supplemented with “T+S” versus untreated UV-killed bacteria on its own (*p =* 0.0196, Fig 7C). There was no difference in apoptosis in animals fed UV-killed versus living bacteria containing “T+S” (*p =* 0.9952).

**Fig 8:**
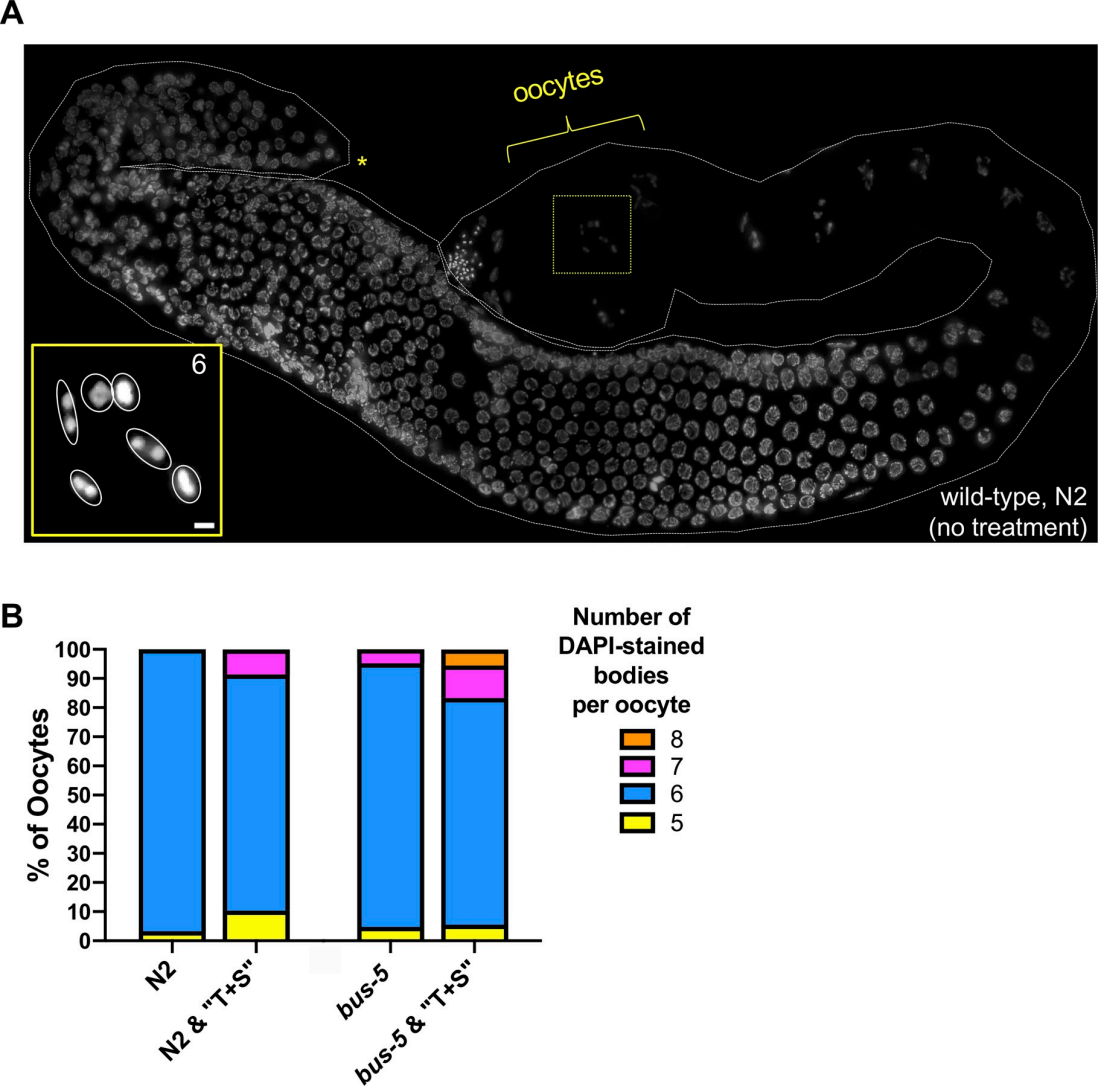
Chromosomal abnormalities in oocytes of “T&S”-exposed *C*. *elegans*. (A) DAPI-stained germ line from wild-type (N2) animal. Yellow brackets indicate region scored for DAPI-staining bodies, which corresponds to late-diakinesis stage of prophase I (i.e. oocytes immediately adjacent to the spermatheca). Asterisk denotes distal tip of gonad. Inset (bottom left) is a projected Z-stack corresponding to the nucleus surrounded by yellow-dashed box. “6” corresponds to the wild-type number of paired chromosomes, which are each outlined in inset in white. (B) Quantification of DAPI-stained bodies in the F1 generation of N2 animals exposed to “T+S” (left) and the P0 generation of the sensitized, cuticle-defective strain *bus-5* (right). Number of gonads scored per genotype are as follows: wild-type (N2) control, n = 30; wild-type (N2) “T+S”, n = 58; *bus-5* control, n = 21; *bus-5* “T+S”, n = 18. Significance was determined by a Fisher’s exact test.

Increased germline apoptosis in animals exposed to environmental toxins is concomitant with defective gamete formation [30]. In *C*. *elegans*, exposure to certain pesticides causes chromosomal abnormalities that manifest in aneuploidy [26,30]. We assessed chromosome structure in oocytes adjacent to the spermatheca, the site of fertilization. At this stage of meiosis, diakinesis, oocyte chromatin is highly compacted, and in wild-type germ lines, all six paired bivalents are readily detected (Fig 8A). In wild-type animals hatched on plates containing “T+S,” defects in chromosome number were observed in 19% of oocytes scored as compared to controls (Fig 8B, *p =* 0.0521). In *bus-5* mutants, which were the most sensitive to “T+S” treatment as assessed by apoptosis (Fig 7), defects in chromosome number were observed in 22% of P0 animals exposed to “T+S” (Fig 8B); however, these data were insignificant versus untreated *bus-5* controls.

**Fig 9:**
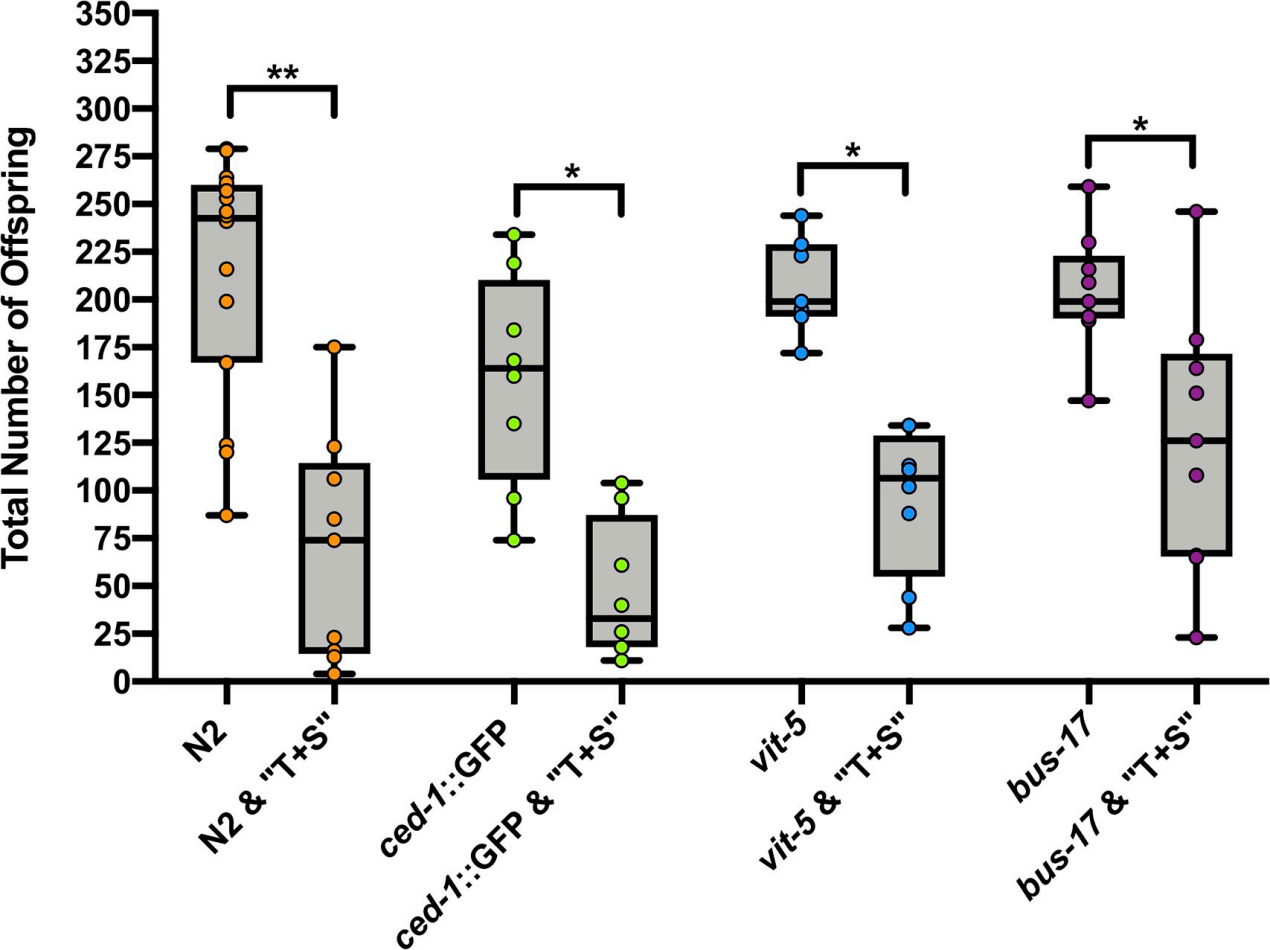
Exposure to “T+S” compromises fertility. Total hatched offspring of wild-type (N2), *ced-1*::GFP, *vit-5*, and *bus*-*17* strains were scored in the presence and absence of pesticide treatment. Each data point (circle) corresponds to total number of F1 progeny from a single animal. Horizontal lines correspond to the median brood size for each data set, and whiskers correspond to range. Total broods scored: wild-type (N2) control, n = 16; wild-type (N2) “T+S”, n = 9; *ced-1*::GFP control, n = 8; *ced-1*::GFP “T+S”, n = 8; *vit-5* control, n = 7; *vit-5* “T+S”, n = 8; *bus-17* control, n = 9; *bus-17* “T+S”, n = 9. Significance was determined by a two-tailed Mann Whitney test. Error bars = S.E.M. *denotes p≤0.01. **denotes p≤0.0001.

Lastly, because germ cell loss can impair fecundity, we compared the brood size of animals in the presence and absence of “T+S”. We scored the total number of hatched offspring laid following “T+S” exposure. A significant reduction was observed in surviving offspring in the F1 generation in both wild-type and sensitized strains (Fig 9).

### “T+S” exposed animals exhibit signs of neural degeneration

**Fig 10:**
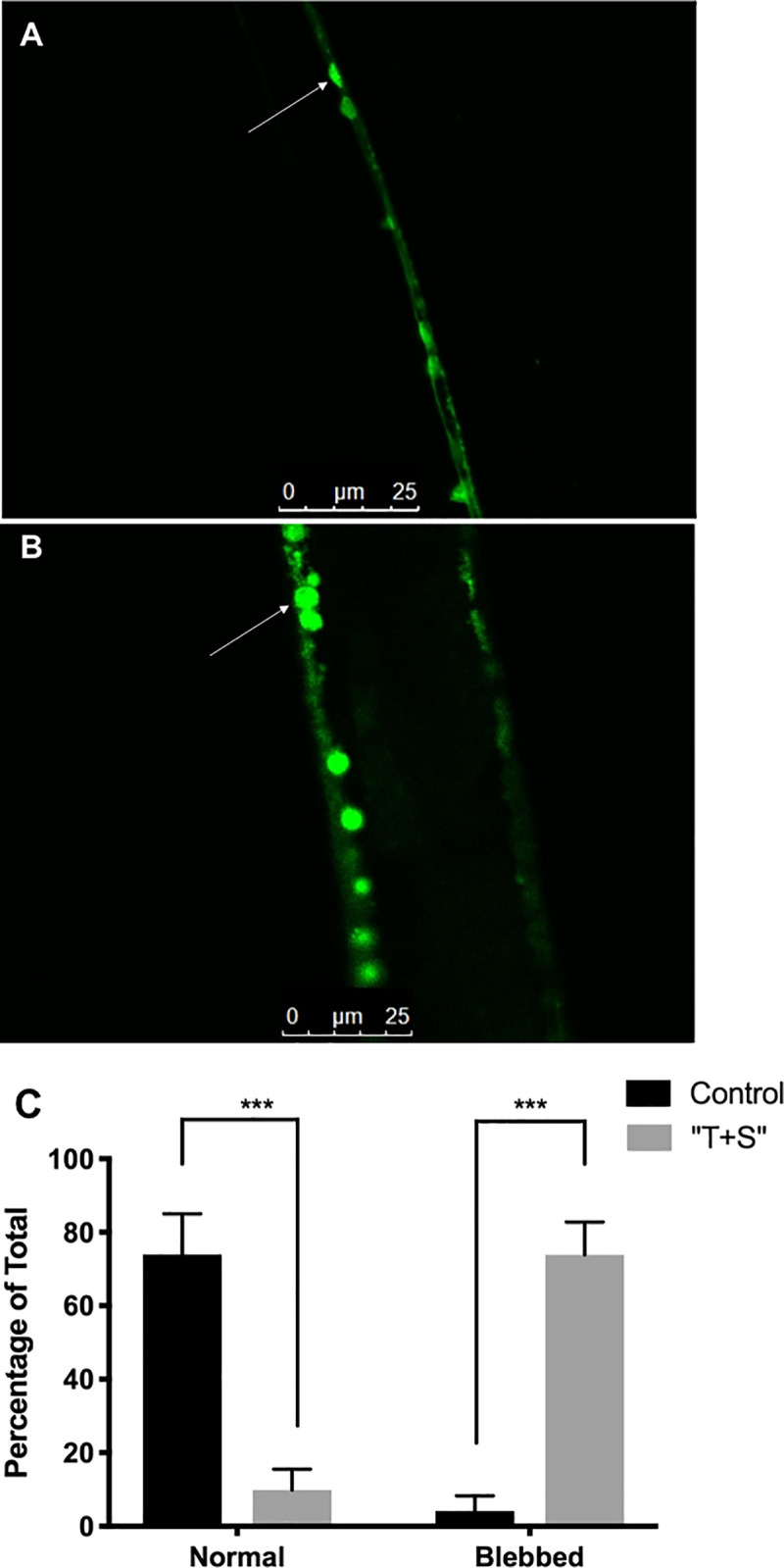
“T+S” exposure leads to neurodegenerative changes in cholinergic neurons. Strain LX929 *C*. *elegans*, which express GFP in all cholinergic neurons, were raised on either control plates (A) or ones with full-strength “T+S” (B), up to L4 stage. *C*. *elegans* were then transferred to agarose gel pads, immobilized with NaN_3_, and imaged through confocal microscopy. (C) Treated worms had a significantly higher percentage of blebbed neurons, a noted sign of aging, than did untreated worms, and untreated worms had a significantly higher percentage of normal, non-blebbed neurons (*p<*0.0001 for both). Indeterminable neurons were excluded from analysis.

Neuronal swelling is a noted sign of neural degeneration [31]. Significantly, more neurons of nematodes raised on 1X “T+S” were rounded in appearance as compared to those of control worms (Fig 10). Of the 89 neurons analyzed from pesticide treated worms, 64 (71.9%) were rounded, 16 (18.0%) were indeterminable, and 9 (10.1%) were not rounded. In contrast, only 1 (4.17%) were rounded, 7 (29.2%) were indeterminable, and 16 (66.7%) were not rounded of the 24 neurons analyzed from control worms (*p<*0.0001).

## Discussion

### “T+S” reduced body size

“T+S,” a commercial insecticide containing 1.47% (w/v) imidacloprid as its active ingredient, is not lethal to *C*. *elegans* when they are exposed on petri dishes. Because we were interested primarily in sublethal effects, we performed our studies using plate-exposed treatment conditions, where the lethality was not significant.

Sublethal exposure to “T+S” impaired growth of *C*. *elegans*, resulting in a reduced body size, as measured by both the perimeter and the area of young adult nematodes. Growth [32] can be affected by impaired feeding or changes in metabolism or expression of key genes involved in growth regulation. We did not see evidence of impaired feeding. The nematodes proceeded through the different larval stages (a food-dependent process) and there was noticeable reduction in the *E*. *coli* present on the patches put on the plates. Growth impairment is a characteristic of metabolic compromise and oxidative damage [33].

It is significant that heat stress, but not paraquat exacerbated the effects of “T+S” to further decrease body size. The fact that paraquat did not exacerbate the effects of “T+S” supports other findings that suggest “T+S” is potentially acting as an oxidative stressor. Our findings suggest that “T+S” could therefore be causing oxidative stress. This increased stress likely leads to even more energy diversion away from growth during development, explaining the overall smaller size of the worms exposed to heat and “T+S”.

### “T+S” decreased locomotion of chronically exposed worms

“T+S” was found to significantly decrease locomotion in nematodes, but only when they were exposed chronically from egg stage and not when they were exposed for 24 hours in the L4, young adult stage. Dorsal and ventral body wall muscles and motor cord neurons finish developing as early as L1 stage [34,35] which suggests that very early development is the most critical time period for normal locomotive development. This could potentially explain why the worms exposed just in their L4 stage were unaffected. Sinusoidal locomotion is modulated by a highly-regulated balance of the excitatory signal induced by the cholinergic motor neurons when they contact muscle cells and the inhibitory signal of the GABAergic motor neurons onto opposing musculature [34–36]. nAChRs are in part responsible for the mediation of excitatory neurotransmission in neurons and muscles, thus it is highly plausible that an nAChR agonist would disrupt this balance. In turn, this could lead to an imbalance of excitatory and inhibitory signaling, thus disrupting normal locomotion.

### “T+S” reduced egg laying

It is significant that “T+S” reduced egg-laying in both worms raised on the compound then transferred to untreated plates to lay eggs, and in worms raised on untreated plates then transferred to “T+S” plates to lay eggs. This suggests that the compound both inhibits egg development and signals to worms they are not in a favorable environment to oviposit.

It is well established in many types of organisms that reproduction slows or ceases in times of low energy availability [37]. Another possible explanation for our results is that the worm’s fertile period was either shifted later or shortened, altering egg production during the observed time period indirectly in this manner. There is a growing body of research examining the role of oxidative stress in development and aging, and several studies have shown oxidative stressors to potentially slow down these processes [38]. If “T+S” is indeed acting as an oxidative stressor and slowing development, worms used in these assays may not have reached full sexual maturity by the time experiments were conducted and may not be producing as many eggs yet, explaining the observed decrease in egg production. Egg-laying behavior involves a regulated activation of cholinergic receptors. An additional mechanism by which the “T+S” reduces egg-laying might result from the unregulated, overstimulation of acetylcholine receptors by imidacloprid’s neonicotinic moiety. More research is needed to explore the mechanisms behind the reduced egg-laying caused by the insecticide exposure.

### “T+S” increased germline apoptosis and reduced brood size

In situations where oocyte quality is compromised, *C*. *elegans* have several strategies to improve reproductive success, one of which is increased germ cell death [39]. Since we did not observe a significant increase in the number of inviable embryos (data not shown), it is likely that a majority of damaged germline nuclei were culled by apoptotic machinery prior to fertilization. It is not known, however, whether additional factors aside from increased apoptosis contribute to impaired fertility. These could include structural defects in oocytes resulting from meiotic errors, as has been documented by exposure to other pesticides known to induce reproductive toxicity during oogenesis in worms and mammals [23,24,40]. Importantly, pesticide-induced reproductive toxicity is not limited to oogenesis, as neonicotinoid insecticides have been shown to significantly compromise sperm function in mice [41]. Further studies will be necessary to determine impacts on *C*. *elegans* spermatogenesis.

Our findings that “T+S” induces germline apoptosis and impedes fertility corroborate recent findings by Shin and colleagues, which establish *C*. *elegans* as a model for assessing negative effects of germline exposure to environmental toxicants [26]. Given that meiosis and germline function are conserved processes known to be hindered by a wide array of environmental toxins, our data warrant future studies to assess mechanistically how “T+S” disrupts gamete formation in worms. As an example, it is unknown whether “T+S”-induced apoptosis is checkpoint-dependent or whether exposure alter germline-specific gene expression. Another intriguing possibility is that exposure to “T+S” could lead to epigenetic changes in gametes that could stably disrupt gene expression patterns in subsequent generations. In support of this, there are extensive examples of environmentally-induced epigenetic inheritance in diverse species including both worms and humans [42].

### “T+S” led to degeneration of cholinergic neurons

Worms raised on “T+S” had significantly more rounded cholinergic cord neurons than did control worms. Swelling of the cell body is an early indicator of neural necrosis [43], and neuron-swelling is an established characterization of neuronal aging in *C*. *elegans* [44]. Further, the manganese-containing pesticide, Mancozeb, led to neurodegenerative change in dopaminergic neurons in *C*. *elegans* [45] and has been linked to Parkinsonism in humans exposed to high or chronic levels of this pesticide [46]. While some degenerative swelling is normal in neurons of aged worms, the process appears to be occurring much earlier and to a much greater extent in “T+S” worms. These age-dependent neuronal defects do not normally appear until the worms are in their post-reproductive stages [44]. In contrast, worms examined in this study were young L4 stage, having not yet reached adulthood or full sexual maturity, yet they still displayed these signs of degeneration.

A similar effect was observed in honeybees exposed to imidacloprid [47]. The authors attributed this swelling to larger intercellular spaces within neurons present in exposed honeybees, likely due to damage in cell-cell contact resulting from alterations in the cytoskeleton. However, the exact mechanism by which imidacloprid lead to this effect was unclear. Additionally, these researchers found decreases in ATP levels resulting from imidacloprid exposure altered the shape of neuronal nuclei and that there was a significant increase in the expression of β-amyloid precursor, a neuronal degeneration indicator protein. Overall, this contributed to significant neuronal damage, which they found to be correlated to deficiencies in behavior of exposed honeybees. Thus, it is probable that the swelling observed in the cholinergic neurons of *C*. *elegans* could result in the observed behavioral effects as well.

## Conclusions

The findings of this study suggest that environmentally relevant concentrations of “T+S” exert damaging effects on nematodes, impacting their development, locomotion, and egg-laying behavior, as well as meiosis. While it remains possible that some of the effects we observed were a result of the pesticide affecting bacteria, the nematodes’ food source, we believe this is unlikely because the treatment was not lethal to nematodes and they were able to grow, develop to adulthood and reproduce. Further, when fed dead bacteria supplemented with pesticide, an increased number of apoptotic nuclei were observed in the germ line. Therefore, we believe the most likely scenario is that the pesticide mixture exerts sublethal effects on nematodes directly. As a whole, these results address several key areas of environmental and biomedical research, from organismal to environmental toxicology, and warrant further analysis of how “T+S” and other commercial formulations impact non-target organisms and potentially contribute to chronic human disease. Though the environmental and ecological impact of neonicotinoid insecticides remains poorly understood, the findings of this study support others showing similar deleterious effects on organism health exerted by neonicotinoid insecticides.

## Materials and methods

### Alleles and strain maintenance

The following strains were obtained from the *Caenorhabditis* Genetics Center at the University of Minnesota: wild-type Bristol N2, LX929((*vsIs48*)[*unc-17*::GFP] (transgenic cholinergic neuronal reporter)), *ced-1*::GFP (transgenic apoptosis reporter), *bus-*17 (cuticle defective), *bus*-5 (cuticle defective), and *vit*-5 (egg yolk protein). Nematodes were incubated at 20º C on nematode growth media (NGM) petri plates with small lawns of bacterial strain OP50 [48]. Stocks were maintained through transfer of four adult hermaphroditic worms to fresh NGM plates with a lawn of OP50 *E*. *coli* bacteria as a food source every four days using a flame-sterilized flattened platinum wire. Small-scale synchronized cultures were prepared for “T+S” exposure by transferring 10–20 gravid adults to fresh NGM plates with OP50. The adult worms were allowed to lay eggs for approximately 3 hours before they were removed, leaving age-synchronized eggs behind.

### “T+S” exposure

Effects of exposure to “T+S” on young adult (L4 stage) worms exposed both acutely for 24 or 48 hours from L3 stage and chronically (3 day exposure) from egg-stage to adulthood were examined. The L4 life-stage was chosen to encompass the beginning of egg production without significant aging within the worms. Diluted “T+S” solutions were prepared for several initial experiments in distilled water, though the recommended application is the compound at its full strength, so the majority of experiments were conducted using the undiluted compound. Fifty microliters of solution were spread on the surface of 2 cm NGM agar plates using a sterile angled glass rod and the resulting mixture was allowed to soak into the agar for 24 hours before a bacterial lawn of OP50 was applied. All plates were produced from a single stock solution of pesticide, which is not volatile, to control for plate to plate variability in pesticide application. According to manufacturer information, the pesticide mixture is stable over the period used for each experiment. Control plates were prepared the same way, except only nanopure water and bacteria were used. In a second method of exposure, which was used for scoring apoptosis, chromosome structure, and brood size analysis, we fed worms a mixture of equal amounts of OP50 bacteria and “T+S”, which was seeded directly onto the plates, which were then allowed to dry for 48 hours at ambient room temperature. For experiments where animals were fed UV-killed OP50 bacteria, plates were seeded with 100 μL live OP50 *E*. *coli* and were dried at room temperature for 48 hours. Lawns of OP50 were subsequently exposed to UV radiation for 60 min using a Variable Intensity UV Transilluminator (VWR) set to high. 100 μL of “T+S” was pipetted onto control, untreated lawns and UV-killed bacteria lawns and allowed to dry. All prepared plates were stored at 4ºC for no longer then 2 weeks prior to using for experiments. Unless indicated otherwise, all experiments were performed at 20ºC.

### Survival assays

Although the manufacturer claims that “T+S” is not toxic to non-target organisms, this needed to be confirmed before assessing sub-lethal effects of the compound. To do so, the survivorship of day 3 adult *C*. *elegans* was measured 48 hours after exposure to varying concentrations (1X, 0.1X, 0.01X, 0.001X, and 0.0001X) of “T+S”. We also exposed nematodes under different conditions (described above) to discern whether the type of exposure influenced sensitivity to the pesticide.

### Size assays

Time-synchronized eggs were collected as described in the section on “Alleles and Strain Maintenance,” and raised in control conditions for 72 hours, before ten worms were transferred to each plate with “T+S” concentrations of 1X, 0.1X, 0.01X, 0.001X, 0.0001X, prepared as described above, or control plates with a lawn of OP50. There worms were allowed to grow on the plates for 48 hours. The worms were then imaged at 20X magnification using an Olympus SZX12 microscope. Image J/Fiji software was used to trace the worms and determine their area and perimeter.

To determine the exacerbating effects of “T+S” on heat exposure, worms were raised from eggs on 1X “T+S” plates as described above until they reached the L4 stage. On day 5, nematodes were placed in a 35˚C incubator for 8 hours. To determine the effects of paraquat, a well-established oxidative stressor, on the toxicity of “T+S”, worms were raised from egg stage on plates with specified concentrations of “T+S” as described above until they reached the L4 stage. On Day 5, the nematodes were transferred to NGM plates with 0.2 mM paraquat for 8 hours. At the end of these 8-hour exposures, worms were imaged at 20X magnification and measured using FIJI software. All experiments were repeated three times using nanopure water as a control.

### Locomotion measurements

For acute locomotion assays, late stage L4 worms were transferred individually to “T+S”-treated or control plates for 24hrs. These worms were then transferred to control plates for testing. Nematodes were transferred individually with a flame-sterilized flattened platinum wire to a fresh NGM plate without an OP50 food lawn. Worms were given several seconds to acclimate to the plate before full body bends consisting of sinusoidal cycles initiated by head oscillations with full waves propagated along the length of the worm were counted for a thirty-second interval.

For each locomotion assay, “T+S”-exposed and unexposed nematodes were tested on the same day to minimize day-to-day variability in locomotion. All locomotion tests were conducted at ambient temperature (22 ºC) with the lid off the petri plate. Nematodes were examined with an Olympus SZ-1 dissecting microscope at 400X magnification.

### Egg-laying behavior

Single, late-stage L4 nematodes were transferred to “T+S”-treated or untreated plates with OP50 bacterial lawns. Each worm was allowed to lay eggs for 24 hours before the eggs were counted and each worm was moved to a new plate (either treated or untreated with an OP50 bacterial lawn) and allowed to lay eggs for another 24 hours, which were then counted. This was repeated for a third time, providing the worms a total of 72 hours (from the start of their L4 stage) to lay eggs.

This same procedure was then conducted on worms raised from eggs to their L4 stage on “T+S” plates, but allowed to lay eggs on control plates, again for a total of 72 hours.

### Confocal microscopy

*C*. *elegans* strain LX929 (vsIs48[unc-17∷GFP]), which express GFP in all cholinergic neurons, were allowed to lay eggs for one hour on control plates with a lawn of OP50 bacteria, and the eggs were then transferred to 2 cm plates either treated with 1X “T+S” or left untreated. All plates contained a bacterial lawn of OP50 bacteria. Worms were allowed to develop for 90 hours at 20ºC before adult hermaphroditic worms for each of 4 independent trials were transferred to a 3% agarose pad and immobilized in a drop of buffer containing 2.5 mM NaN3. Images were taken using a Nikon PCM2000 confocal laser-scanning microscope equipped with an argon laser.

A total of 113 neurons from 12 different worms exposed to either “T+S” or control conditions were examined. For each neuron analyzed, images were collected using a 40X lens. The percent of cholinergic neurons along the nerve cord that exhibited blebbing and rounding, morphological changes observed in the progression of neuronal degeneration, was quantified for both treated and control worms by blinded participants. Neurons that were of undeterminable classification were discarded from analysis.

### Quantification of germline apoptosis

Acridine orange (hereafter “AO” staining”) and *ced-1*::GFP expression were used to score apoptosis in response to exposure to “T+S”. AO is pH-sensitive and fluoresces in response to a pH change which occurs when the cells are apoptotic [49]. *ced-1*::GFP worms contain a transgene which is expressed when the sheath cell engulfs a cell which has been tagged for apoptosis due to physiological stress or damage to DNA [49,50]. Briefly, animals were staged as L4 larvae and were either assessed as the P0 generation 48 hours later or were allowed to develop and lay embryos for 4 days before F1 offspring at the L4 larval stage. Animals were then picked to fresh plates for AO staining or directly to 3% agarose pads for quantification of CED-1::GFP, as described above. Experiments comparing the same generation/genotype with and without “T+S” treatment were performed synchronously, and data presented are compiled from multiple experiments. A minimum of 32 gonads were scored per condition. For all assays, apoptotic bodies were imaged using a Leica DM5500 automated upright fluorescent microscope and were quantified as described in [51].

### Brood size analysis

To assess fertility, wild-type N2, *ced-1*:*GFP*, *bus-17*, and *vit-*5 worms were staged as L4 larvae and picked 1 worm per plate. Brood size analysis was conducted as described in [52]. Briefly, L4 hermaphrodite worms were transferred to new plates approximately every 24 hours over the course of 3 days. Total hatched progeny on each plate were counted 2–3 days after being laid. All strains were assessed in parallel and data were pooled from multiple rounds of analyses, with a minimum of 3 per genotype.

### Cytological analyses

Animals were staged as L4s and dissected 48 hours later. Removal of gonads was performed in egg buffer supplemented with 0.1% Tween, followed by fixation in 5% paraformaldehyde. Dissections were performed as described in [53] with the following modifications: freeze-crack was performed after a minimum five minute incubation of charged slides on an aluminum block at −80°C. After freeze-crack of coverslips, slides were immersed in −20°C methanol for one minute and washed in 0.1% PBS Tween. Slides were mounted using EMS Shield Mounting Medium with 4,6-diamidino-2-phenylindole (DAPI) and DABCO™ (Electron Microscopy Sciences) and sealed with nail polish. Images were captured using a Leica DM5500 automated upright fluorescent microscope and Leica Application Suite X (LAS X). Microscopy data were processed and analyzed using Image J open source software (Wayne Rasband, NIH).

### Statistical analyses

All statistical analyses were conducted using Prism (GraphPad Software, LLC). A p-value of less than 0.05 was considered statistically significant. For survival assays, the percent surviving for ten independent trials at each concentration was analyzed was analyzed using a 2-way ANOVA and pairwise *t*-tests with Bonferroni correction. For developmental assays, individual worms were measured, and data were analyzed by one- way (6 concentrations of T+S) ANOVA for “T+S” impaired growth and by two-factor (concentration x treatment) ANOVA for studies examining the synergistic effects of “T+S” and either heat or paraquat exposure. When the ANOVA detected statistical significance, Tukey’s multiple comparison test was used to determine where the significance lay.

To determine the effects on “T+S” on locomotion, body bends of individual worms were counted and pair-wise t-tests were used to evaluate significance. To analyze the effects of “T+S” exposure on egg-laying behavior, eggs from individual worms were counted and analyzed using one-way ANOVA. To quantify the effect of “T+S” exposure on cholinergic neurons, the percentage of swollen neurons was determined for both control and treated worms, and a one-way ANOVA was used to determine statistical significance.

Two-tailed Mann Whitney tests were used to analyze germline apoptosis and brood size. A Fisher’s exact test was used to assess presence of chromosomal abnormalities in DAPI-stained germ lines.
